# Immunoglobulin G4-related disease presenting with peripheral neuropathy: a case report

**DOI:** 10.1186/s12883-021-02069-z

**Published:** 2021-01-28

**Authors:** Jeong Bin Bong, Dong Kun Lee, Min A Lee, Byoung Wook Hwang, Hyun Goo Kang

**Affiliations:** 1 Department of Neurology, Chosun University College of Medicine, Gwangju, South Korea; 2 Department of Neurology and Research Institute of Clinical Medicine of Jeonbuk National University - Biomedical Research Institute of Jeonbuk National University Hospital, 20 Geonji-ro, Deokjin-gu 54907 Jeonju, South Korea

**Keywords:** IgG4-related disease, Inflammatory neuropathy, Asymptomatic tumor‐like lesion

## Abstract

**Background:**

Immunoglobulin G4-related disease (IgG4-RD) is an immune-mediated fibro-inflammatory condition characterized by high serum IgG4 concentrations and tissue infiltration by IgG4-positive plasma cells. Reports have demonstrated that IgG4-RD affects various organs, including the pancreas, kidney, lung, thyroid, and lacrimal and salivary glands. In the nervous system, hypertrophic pachymeningitis and hypophysitis are mainly related to IgG4-RD; however, the peripheral neuropathy involvement is unusual.

**Case presentation:**

We report on a 69-year-old woman with multiple mononeuropathy, weight loss and kidney mass in the setting of IgG4-RD. Biopsies of the kidney mass showed lymphoplasmacytic sclerosing inflammation with numerous IgG4-positive plasma cells. IgG4 and IgG4/IgG ratios in the blood were elevated. The patient was treated with high dose methylprednisolone with improvement in her neuropathy.

**Conclusions:**

IgG4-RD is a relatively recently reported systemic fibrous inflammatory disease caused by the infiltration of IgG4-positive plasma cells in various organs. In the nervous system, symptomatic peripheral nerve invasion is very rare. However, as demonstrated in our case, IgG4-RD may present with primarily peripheral nerve disease.

## Background

Immunoglobulin G4-related disease (IgG4-RD) is an immune-mediated fibro-inflammatory disease. Histologically, it is characterized by the infiltration of IgG4-positive plasma cells, storiform fibrosis, and obliterative phlebitis in the involved organs, with an increase in serum IgG4 [1]. It was first reported in patients with autoimmune pancreatitis [2]. It was later found that IgG4-RD is a systemic disease which may affect other organs throughout the body. Organs most affected are the pancreas, kidneys, lungs, and the retroperitoneum [3]. In the nervous system, hypertrophic pachymeningitis and hypophysitis may occur, but invasion of the peripheral nerves is unsual. We encountered a patient with IgG4-RD, presenting with neuropathy. We report this case with a review of the literature.

## Case presentation

A 69-year-old woman presented with a 2 months history of progressive right hand weakness. A year prior, she was diagnosed with hypertension and diabetes. The patient had lost 8 kg unintentionally and had been experiencing generalized weakness in the six months prior to presentation. She frequently dropped objects and could not use a cooking knife because her right hand was weak. These challenges had lasted for approximately two months. Her condition gradually deteriorated over a two-week period to the point where she could not use chopsticks. Upon examination, she had right hypothenar and first dorsal interossei muscles atrophy. She had weakness in the right fingers abduction and adduction (Medical Research Council [MRC] grade 3) and the left ankle dorsiflexion (MRC grade 4). Hypesthesia was observed in the 4th and 5th fingers of the right hand and the medial part of the palm. The rest of her examination, including deep tendon reflexes were normal.

Nerve conduction studies and electromyography are summarized in the table. (Tables 1 and 2). Our impression was that of a multiple mononeuropathy, affecting the right ulnar and left deep peroneal nerves. Additionally, needle electromyography revealed abnormal spontaneous activities in the right first dorsal interossei and abductor digiti minimi muscles as well as in the left tibialis anterior muscle. The motor unit action potential recruitment and interference pattern also decreased. However, abnormal spontaneous activities were not observed in the left tibialis posterior and tensor fasciae latae muscle, and left peroneus longus muscle, respectively. Therefore, lumbar radiculopathy (mainly L5) and left common peroneal neuropathy could be excluded.

**Table 1:** Results of nerve conduction study

	Right/Left				Right/Left	
	Latency (ms)	Amp (mV)	NCV (m/s)		Amp (uV)	NCV (m/s)
Motor NCS				Sensory NCS		
Median nerve				Median nerve		
Wrist	3.1/3.0	11.7/12.0	-	Finger-wrist	20.0/24.1	41.1/41.2
Elbow	7.1/6.9	11.6/11.7	55.0/52.1	Wrist-elbow	32.3/41.0	50.0/51.1
Axilla	8.4/8.3	11.0/11.2	57.7/53.0	Elbow-axilla	121.7/119.8	53.6/52.5
F-wave	-					
Ulnar nerve				Ulnar nerve		
Wrist	2.4/2.3	**2.2**/8.3	-	Finger-wrist	**8.2**/22.0	44.4/44.0
Below elbow	6.3/6.0	**2.3**/8.7	51.3/56.8	Wrist-elbow	14.5/26.4	48.5/48.8
Above elbow	8.3/8.0	**2.1**/8.2	50.0/50.0	Elbow-axilla	22.8/36.8	50.0/59.1
Axilla	8.9/8.6	**2.1**/8.3	50.0/50.0			
F-wave	**34.4**/27.9					
Peroneal nerve				Superficial peroneal nerve	9.8/11.9	40.0/37.6
Ankle	3.1/5.2	2.2/**0.5**	-			
Knee	10.3/12.5	2.1/**0.3**	42.5/**38.2**			
F-wave	-					
Tibial nerve				Sural nerve	8.1/8.5	37.0/40.9
Ankle	4.1/3.6	9.8/10.1	-			
Knee	13.6/12.7	9.7/10.1	42.3/43.5			
F-wave	48.8/49.0					

*Amp* Amplitude, *NCV *Nerve conduction velocity, Abnormal values are underlined

**Table 2:** Results of needle electromyography

Muscle	Spontaneous activity	Motor unit morphology	Interference pattern
Right first dorsal interosseous	Fibs and PSWs	Normal	Reduced
Right abductor digiti minimi	Fibs and PSWs	Normal	Reduced
Right abductor pollicis brevis	Normal	Normal	Normal
Right flexor digitorum profundus (digit 4,5)	Normal	Normal	Normal
Right flexor carpi ulnaris	Normal	Normal	Normal
Right pronator teres	Normal	Normal	Normal
Right extensor digitorum communis	Normal	Normal	Normal
Right biecps brachii	Normal	Normal	Normal
Right cervical paraspinals	Normal		
Left tibialis anterior	Fibs and PSWs	Normal	Reduced
Left peroneus longus	Normal	Normal	Normal
Left tibialis posterior	Normal	Normal	Normal
Left gastrocnemius	Normal	Normal	Normal
Left vastus lateralis	Normal	Normal	Normal
Left biceps femoris short head	Normal	Normal	Normal
Left tensor fasciae latae	Normal	Normal	Normal
Left lumbar paraspinals	Normal		

*Fibs* Fibrillation potentials, *PSWs* Positive sharp waves

Blood tests showed leukocytosis (25,120/mm^3^; normal range 4,000–10,800/mm^3^); neutrocyte 86.8 %(normal range 40–80 %) and lymphocyte 6.1 %(normal range 25–50 %), thrombocytosis (692,000/mm^3^; normal range 150,000–400,000/mm^3^), and hypochromia (8.5 g/dL; normal range 12–16 g/dL). The erythrocyte sedimentation rate was increased to 120 mm/hr (normal range 0–30 mm/hr). The results of the serum test showed a slight increase in total protein (7.53 g/dL; normal range 5.3–7.4 g/dL) and a decrease in albumin (2.54 g/dL; normal range 3.5–5.2 g/dL). A reversed albumin/globulin ratio (0.51; normal range 1.0–2.0) and an increased C-reactive protein (5.61 mg/dL; normal range 0.0–0.3 mg/dL) were observed. Anti-myeloperoxidase and anti-proteinase 3 antineutrophil cytoplasmic antibodies (ANCA) were negative. Electrolyte, liver function, and renal function tests were normal. Serum protein electrophoresis showed multiclonal gammopathy. Serum immunofixation electrophoresis was normal.

**Fig. 1:**
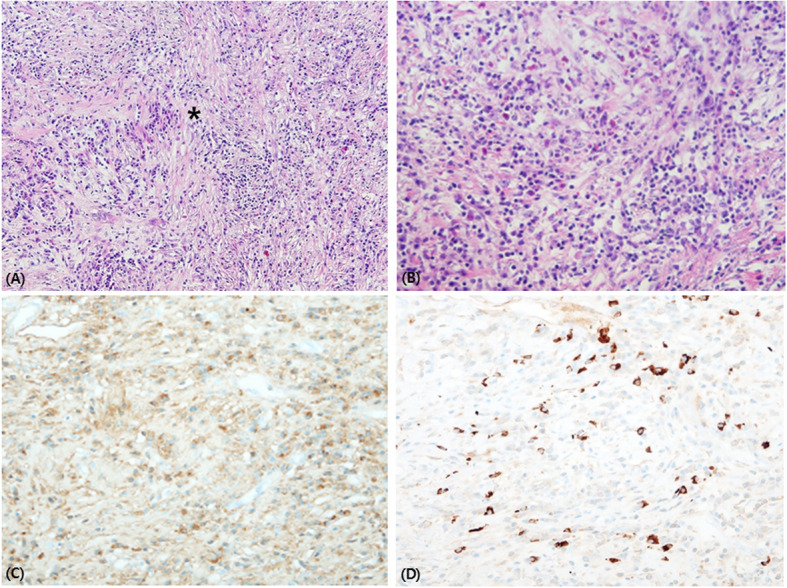
Pathologic findings. **a** Kidney biopsy showed inflammatory cell infiltration with storiform fibrosis (asterisk). **b** The inflammatory cells are predominantly plasma cells and lymphocytes. H&E statin, (A) x100, (B) x200. The immunohistochemical stain (x200) for IgG (**c**) and IgG4 (**d**) revealed numerous IgG + and IgG4 + plasma cells (brown color) and an increased ratio of IgG4+/IgG+ (64.9 %)

Chest and abdominal computed tomography were conducted to identify the causal factors of the elevated inflammatory index. A tumor 7.5 cm in size was observed in the right kidney, and a renal biopsy was performed to rule out malignancy. The results of the histopathological examination showed fibrosis accompanied by considerable infiltration of plasma cells, and IgG and IgG4 immunohistochemical staining revealed an IgG4/IgG ratio of 64.9 % (Fig. 1). A serological test was also performed, and the results showed an IgG concentration of 1551 mg/dL (normal range 870–1700 mg/dL) and an IgG4 concentration of 177.8 mg/dL (normal range 11–157 mg/dL), with an IgG4/IgG ratio of 11.5 %. She was diagnosed with IgG4-RD based on the clinical course and test results and treated with intravenous methylprednisolone (0.6 mg/kg daily) for 10 days followed by oral methylprednisolone at 10 mg/day for maintenance therapy. The right hand weakness improved two weeks after initiating steroid treatment, and she could use chopsticks again. Her general weakness also gradually improved. However, she still had discomfort while walking on her left heel. On follow-up examination after 3 months, her serum IgG4 concentration was within the normal range (54.5 mg/dL). Thereafter, the prednisolone treatment was tapered to 5 mg/day for maintenance therapy and she was started on methotrexate 7.5 mg/week.

## Discussion and conclusions

Our patient visited the hospital because of weakness of the right hand in the setting of weight loss and generalized weakness. Electrophysiological tests showed multiple mononeuropathy. Blood tests suggested systemic inflammation. A right kidney tumor-like lesion was found on abdominal computed tomography. Eventually, IgG4-RD was diagnosed using histopathology and serological tests.

IgG4-RD is a systemic fibrous inflammatory disease that has been recently reported. In early 2001, increased serum IgG4 was observed in a patient with autoimmune pancreatitis, and IgG4-positive plasma cells were observed in the immunostaining of pancreatic tissue [2]. Kamisawa et al. proposed an IgG4-related autoimmune disease as a systemic disease caused by the infiltration of IgG4-positive plasma cells in various organs such as the biliary tract, salivary glands, lacrimal glands, liver, kidney, lung, and retroperitoneal cavity in addition to the pancreas [3]. The disease was named IgG4-RD by the International Multidisciplinary Research Group in 2012, and the name has been in use since then [4]. In the nervous system, hypertrophic pachymeningitis, pituitary invasion, and orbital diseases have been mainly reported; cerebral parenchymal and peripheral nerve invasions have also been reported, albeit rarely [5]. In the case of our patient, peripheral neuropathy was first identified, and an asymptomatic right renal tumor-like lesion was found incidentally during the evaluation of the causal factors of the disease. IgG4-RD was diagnosed by renal histopathology and serological tests.

IgG4-related neuropathy can be misdiagnosed as vasculitic neuropathy, particularly vasculitis associated with ANCA. Therefore, a differential diagnosis is required. Ohyama et al. reported that when IgG4 immunostaining was performed for 149 patients who were diagnosed with inflammatory peripheral neuropathy through nerve biopsy, 29 of them had sufficient IgG4-positive plasma cell infiltration to meet the diagnostic criteria for IgG4-RD; 22 of them had been diagnosed with ANCA-associated vasculitis (11 had been diagnosed with microscopic polyangiitis, and 11 had eosinophilic granulomatosis with polyangiitis) [6].

Inoue et al. retrospectively evaluated 106 patients with IgG4-RD and found 21 peripheral nerve lesions in seven patients [7]. Invasion of the orbital nerves, optic nerves, and spinal nerves was found mainly in the orbital and paravertebral regions. Although patient condition was neurologically asymptomatic at the time of onset, loss of vision due to recurrent lesions around the optic nerve was reported in one case. There was only one patient that had peripheral nerve invasion only. This was confirmed by a sural nerve biopsy, which showed a good response to steroid treatment [8]. 

We believe this our patient has IgG4-RD manifesting as multiple mononeuropathy, weight loss and malaise. Our case is supported by the fact that the neurological symptoms as well as the serum IgG4 concentration improved with steroid treatment and by the fact that we rule out other causes of neuropathy. A nerve biopsy would have even supported our hypothesis further but was not performed due to patient’s wishes. In summary, physicians should consider IgG4-RD in patients with acute/subacute neuropathy in the setting of systemic symptoms and evidence of elevated serum IgG4 and/or IgG4-positive plasma cells in various organs.

## Data Availability

All data and material supporting our findings are contained within the manuscript.

## Abbreviations

IgG4-RD: Immunoglobulin G4-related disease
ANCA: Antineutrophil cytoplasmic antibodies
MRC: Medical Research Council
